# Developing a measure to assess the quality of care transitions for older people

**DOI:** 10.1186/s12913-019-4306-8

**Published:** 2019-07-19

**Authors:** Eirini Oikonomou, Eleanor Chatburn, Helen Higham, Jenni Murray, Rebecca Lawton, Charles Vincent

**Affiliations:** 1 University of Oxford, Oxford, UK; 2 University of Bath, Bath, UK; 3 Yorkshire Quality and Safety Research Group, Bradford Institute for Health Research, Bradford, UK; 4 School of Psychology, University of Leeds, Leeds, UK

**Keywords:** Transitions, Measure, Patient safety, Questionnaire, Older patients

## Abstract

**Background:**

The transition of older patients (over 65 years of age) from hospital to their own home is a time when patients are at high risk. No measure currently exists to assess the experience, quality and safety of care transitions relevant to UK population. We aim to describe the development and initial testing of the Partners at Care Transitions Measure (PACT-M) as a patient-reported questionnaire for evaluation of the quality and safety of care transitions from hospital to home in older patients.

**Methods:**

We used an established measure development procedure which includes conceptualising the components of care transitions, item development, conducting a modified Delphi process and pilot-testing of the PACT-M with patients over 65 years old using telephone administration.

**Results:**

Pilot testing of the PACT-M suggests that the components identified cover the issues of most importance to patients. Face validity testing showed that the measure in its current form is acceptable to older patients.

**Conclusions:**

The measure developed in this study shows promise for use by those involved in planning, implementing and evaluating discharge care, and could be used to inform interventions to improve the transition from hospital to home for older patients.

**Electronic supplementary material:**

The online version of this article (10.1186/s12913-019-4306-8) contains supplementary material, which is available to authorized users.

## Background

Care transition is defined as a series of pre- and post-hospital discharge activities which aim to ensure the coordination and continuity of care for patients who transfer across healthcare settings [1]. The transition of patients from hospital to the post-hospital setting is particularly critical for older patients, especially those with complex care needs [2, 3]. There is an increased risk for health and care problems to occur in the immediate post-discharge period potentially leading to an unplanned readmission within 30 days [2]. The difference between planned and unplanned readmissions is that the latter has been associated with poor discharge care and could be preventable [4] even though some unplanned readmissions are justified by the treatment of a complication [5]. As many as one in five older patients experience an adverse event in the transition from hospital to home, of which 62% are preventable [6, 7].

### Transition from hospital to home

In the United Kingdom, the transition of a patient from hospital to home is a highly variable and complex process that depends on the needs of the patient, their support network, levels of frailty and co-morbidities as well as access to health and social care resources [8]. Most transitions require coordination and communication between many different people: the hospital team, the General Practitioner (GP), community nurse, social worker, family and patient [9].

Transitions between hospital and home may be managed in different ways in different settings in the UK. Ideally, older people and their families will be prepared for transition with information on medicines, recommended care of their condition, action in the event of deterioration in discussion with discharge coordinators and nursing staff. A discharge letter will be sent to their primary care physician but may not arrive until one or two weeks post discharge. Then, contact is made with practitioners from the community or social care services before discharge, an ongoing care plan is agreed, and practitioners’ details are recorded on the discharge plan. After being discharged from hospital, the patient and/or their carer are supported by practitioners in their community multidisciplinary team to manage their condition [10].

Research has shown that patients and their caregivers often do not feel prepared to take on their own care after discharge [11] and struggle to understand key aspects of how to manage their condition and symptoms [12]. They may not know how to contact health professionals for advice [13, 14] and feel frustrated by poor preparation for their discharge home [11]. Care can be fragmented and patients may be uncertain about who is coordinating their care needs [15–17]. An international survey of patients with complex care needs reported that one in four did not receive instructions for follow-up nor did they receive clear medication directions [18].

Strategies to improve transitions have shown mixed results. Rennke and colleagues [18] identified a variety of different approaches to improve transitional care with no consistent evidence for the efficacy of any particular strategy. There is some evidence that interventions to improve transitions can lower readmissions rates, but no effect has been found on improvements to patients’ self-reported quality of life. Recent reviews [19–23] have suggested that interventions to increase patient involvement at transitions show most promise. As these interventions contain multiple components, however, it is not possible to identify which are the most effective aspects.

### Existing measures of care transitions

The Care Transition Measure (CTM) [24] is currently the primary means of assessing the quality of the transition between hospital and home. The CTM, developed in the US, includes questions on information transfer, patient and caregiver preparation, self-managing medication and empowerment to assert preferences. Although the CTM is a valuable measure, we considered that it would be necessary to develop a new measure. The UK healthcare system is very different from the United States and it is likely that patients and families face different challenges. We also wished to explore whether there were other facets of the transitions, particularly safety related, that are not represented in the CTM. Finally, the CTM focusses on the immediate post-discharge period whereas we wished to examine both the immediate as well as the longer term experience of care after the patient returned home. Our own work identified that knowing and understanding things at the point of discharge does not necessarily translate into longer-term confidence in ‘self-care’ on returning home.

Other measures of care transitions have addressed care co-ordination, continuity, patient satisfaction and quality assurance [15, 25–33]. However, all these measures have a very specific focus on care delivered at a particular site – such as the hospital, nursing home, or primary care clinic – rather than the transition between hospital and home. Many of these authors call for further studies to explore the core factors underpinning quality of care transitions.

### Aims of the present study

The purpose of the present study was to develop a measure of care transitions that could assess patient experience of the transition and thereby be a marker of quality of transitional care. The specific objectives of the study were:

- To develop a framework of core components of the transition from hospital to home.
- To develop a measure to evaluate the quality and safety of care transitions relevant to older patients.
- To pilot this measure with older patients 65 years of age or older to make an initial assessment of usability and face validity.

This study is part of a larger programme of work (NIHR RP-PG-1214-20,017 PACT) which seeks to achieve a better understanding of both patient [34] and staff views [35] of care transitions and ultimately to develop an intervention to improve the transition process. This intervention will be evaluated in a randomised controlled trial and assessed by means of the measure currently in development.

## Methods

### Overview of the process of development

**Fig. 1:**
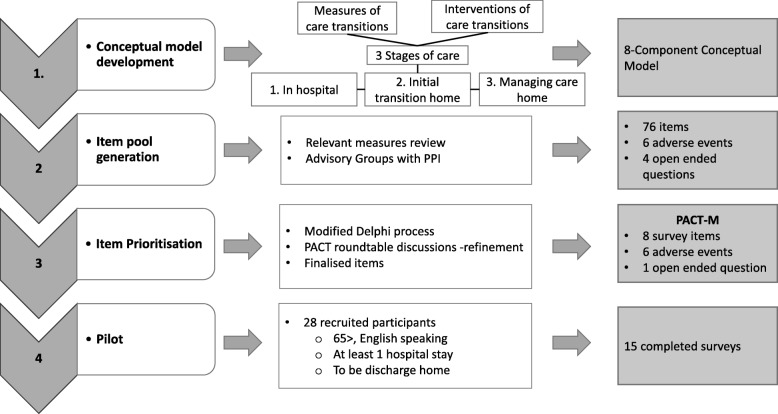
Flowchart of the measure development process

We conducted this study in four stages (Fig. 1) following the scale development procedure described by DeVellis [36]. Our first task was to (1) develop a conceptual model. We started by defining the time period of interest and the type of transition relevant to this particular programme and subsequent intervention. We then defined the core themes of transition in the sense of conceptualising the critical aspects of the experience of the transition process. We did this through a literature review on existing measures of care transitions, transition interventions, and emerging findings from qualitative studies of both patient and staff experience carried out in an earlier part of the PACT research programme. Secondly, we carried out a thorough process of (2) item generation followed by refinement and simplification of items using a (3) modified Delphi process and feedback from patients and their families. Finally, we refined the language of the measure and carried out (4) pilot testing with 15 patients. In this paper we report on initial data from the pilot questionnaire administration. The complete psychometric evaluation will be reported elsewhere.

### Stage 1: Development of a conceptual model

For the purposes of this project, we defined care transitions as a patient’s journey from hospital to their own home. The PACT measure was designed to assess the discharge experience and how the person concerned, is managing at home at later time points. We excluded transfers from hospital to nursing or residential homes or any other setting in which the primary responsibility for care rests with healthcare professionals rather than the patient and family.

The planned intervention in the wider PACT research programme aims to improve the quality and experience of transition through increased patient involvement but also to support patients in the ongoing management of their care. We therefore decided to orient the PACT-M to address the below three time points of care:

- What was the patient’s experience of preparation in hospital for their return home? This was to be assessed (1) within the first week of discharge when memories were fresh (Time 1).
- How well is the patient managing their care once back at home? This was to be assessed at (2) one month (Time 2) and (3) three months post discharge (Time 3).

### Literature review

We searched PubMed and EMBASE databases for relevant studies between 1995 and 2018 using search terms such as: ‘care transitions’, ‘continuity of care’, ‘measure’, ‘questionnaire’, ‘quality’, ‘safety’, ‘post-discharge’, ‘home care’, ‘patient experience’, ‘interventions’ and ‘adverse events’. The search encompassed studies of measures relevant to care transitions and intervention studies. In parallel with this, we carried out a brief literature review of adverse events in ambulatory settings to identify the most common adverse events encountered.

### Preparatory qualitative studies

We reviewed findings from an earlier qualitative interview study exploring the experience of older people and their families during the transition from hospital to home [34]. Initial insights from qualitative accounts highlighted the key themes that relate to patient experience.

### Patient and carer engagement and feedback

To gain insights into patients’ and carers’ transition experience we involved a Patient and Public Involvement (PPI) panel in the measure conceptualisation. Our PPI panel included patients and carers who use health services and have been through a transition process. We invited people to join two advisory groups and talk about their experiences with healthcare, review our research plans, and help refine the measure aims in ways that can be understood by everyone.

### Identifying core components of a transitions measure

Once we had completed the steps outlined above, we then combined and triangulated evidence from the literature review, research findings of the wider PACT research programme and reflections from our patient panel to define the core components of a measure of patient experience of transition.

### Stage 2: Item pool generation

Item generation proceeded in four phases. First, we used our previous literature review and input of the PPI panel to generate a long list of items relevant to components of care transitions. Second, duplicates were removed and items reviewed for relevance by the wider research team and refined to a shorter list. Third, items were reviewed by our PPI panel for language and level of comprehension. Finally, a modified Delphi process was employed to prioritise items based on relevance and content validity with input from experts in patient involvement, patient safety and transitions including psychologists, sociologists, healthcare researchers and gerontologists.

### Stage 3: Prioritisation of items for inclusion in the measure; a modified Delphi method

We used a modified Delphi method (adapted from [26, 37] to review the item pool and reach consensus on the components and individual items. A ‘mainstream’ Delphi process includes face to face discussions between experts for two or more rounds. The process is stopped after an agreed criterion; for example, after a number of rounds or when consensus is reached [38]. Our modification included two round-table discussions with an expert panel and a web-based Delphi survey to examine content validity of the draft measure, run by the researcher EO. The combination of two review methods has been suggested as the optimal consensus process for item generation [37]. The expert panel was identified through the PACT team (psychologists, healthcare researchers, social scientists and clinicians) and regional networks and expertise was defined as having considerable clinical or managerial experience in acute geriatric care. A total of 25 experts were invited to participate in an on line survey of which, 15 provided feedback on the first round and 22 responded in the second round. Data collection from the on line Delphi survey was undertaken using the Qualtrics survey software.

### Deciding on an appropriate scale

Considering the large burden of illness of the targeted population, as well as the mode of delivery (telephone), all items were designed to be brief and simple using a Likert scale. The ideal number of response options is widely recommended to be between 4 and 7 [39], with 4 or 5 being preferred for telephone administration [40]. We chose a 5-point Likert scale to retain a mid-point response.

### Stage 4: Pilot testing of the PACT-M

#### Pilot size calculation

Survey guidelines suggest that an optimal pilot sample should be 10% of the sample projected for the larger parent study [41, 42] or between 10 and 30 participants for survey research in clinical settings [43–46]. We aim to run a companion study to establish the validity and reliability of the PACT-M for which we aim to recruit 150 participants. Thus, for this pilot study we sought to collect 15 (10% of the larger sample) completed questionnaires.

Piloting work was undertaken at a large National Health Service (NHS) Teaching Hospital. Participants were given a written information document and consent form. Participants were contacted by phone 3–7 days after discharge from hospital and the PACT-M Time 1 questionnaire (for administration within one week post discharge) was administered.

### Participants and recruitment

Participants were 65 years of age or older who had spent at least one night in hospital and were due to be discharged to their own home. Participants had to be English speaking and able to provide informed consent. We excluded people with cognitive impairment.

We recruited 28 patients from medical, surgical, cardiovascular and complex medical care units using opportunistic sampling. Initial screening and eligibility assessment was performed by the clinical team that identified potential participants and introduced them to the researcher on the wards.

Patients that agreed to take part in the study, were contacted by a member of the research team within a week after they have been discharged and asked to complete the PACT-M over the phone.

### Instrument administration

Participants were presented with the PACT-M1 pilot version (please see the Additional file 1) that contained; (a) eight statements participants were requested to rate on a 5-point Likert scale, (b) a list of adverse events to answer with yes or no and (c) two open ended questions. During the phone administration, the researcher prompted participants to comment on the questionnaire structure, duration of the phone call or individual item phrasing. During the administration of the PACT-M1, participants were asked to comment on any difficulties encountered and to make suggestions if they wished on the wording of the questions. After completing the questionnaire they were asked questions around perceived barriers to questionnaire completion and the researcher clarified any questions that may have arisen. These telephone calls lasted from 10 to 25 min and the researcher kept notes on participants’ feedback.

## Results

### Stage 1: Development of a conceptual model

#### Literature review

Data extraction of the relevant papers revealed the following themes: discharge planning, preparation for self-management, patient involvement with own care, information transfer between providers, information transfer to patients and their carers and patient experience across the transition.

We then extracted items from 10 existing measures relevant to our transitions approach [15, 25–33] to ensure that all relevant themes had been included.

### Preparatory qualitative studies

After reviewing a qualitative interview study of the PACT research programme, exploring the transition experience of older people from hospital to home [34] the following key themes were extracted: caring of ward staff, disappointing discharge, difficulties in managing medicines, loss of autonomy and independence during the hospital stay, unmet communication and information needs and lack of receipt by services in the community. A common thread through these themes was lack of patient involvement, whether desired or otherwise.

This qualitative study also identified that one of the key contributing factors for people feeling safe was related to having enough information around medications as people often did not feel confident asking questions about new medication regimes or were reluctant to take them after they have returned home. Information about obtaining healthcare supplies emerged as another prevalent safety issue as patients felt confused around how to get essential equipment which impacted on both their independence and mobility.

### Patient and carer engagement and feedback

Discussions with people participating in the study’s advisory groups emphasised that the PPI panel's experience and knowledge changed throughout the transition process. Dividing the measure into clearly labelled sections – ‘During my stay in hospital’ and ‘After I left hospital’ – was thought to be critical in capturing the transition process. PPI participants confirmed the importance of all themes previously identified, but added that the patient experience of safety should also be assessed. We had provisionally planned to administer the measure by telephone; the panel confirmed that this was acceptable provided that calls were reasonably short and people were informed of how long the phone call would last. Overall, the advisory groups highlighted the need for item phrasing to be clear and focussed on the needs and experience of older people.

### Identifying core components of a transitions measure

Once we had completed the steps outlined above we then combined and triangulated evidence from the literature review, research findings of the wider PACT research programme and reflections from our patient panel to define the core components of a measure of patient experience of transition.

### Defining core components

**Fig. 2:**
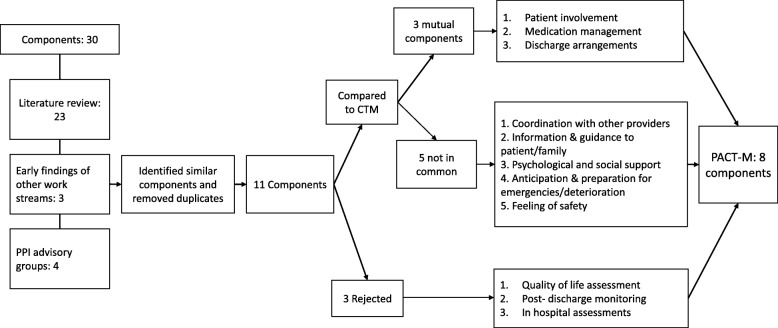
Flowchart of component classification

After consolidating evidence and findings from the review stages, we constructed a framework composed of 30 potential components (Fig. 2); by removing duplicates we were able to reduce the list to 11 overarching components. Three of these were eliminated because they were beyond the scope of a measure founded on the patient experience of transitions. These were: (i) quality of life assessment, which was important but best assessed using one of the many existing measures, (ii) clinical assessments and findings in hospital, which would not necessarily be available to patients and (iii) post-discharge monitoring of mortality as we aimed not to contact families where patients had died.

The remaining 8 components were conceptualised as:

1. Patient involvement
2. Medication management
3. Discharge arrangements
4. Coordination with other providers
5. Providing information and guidance to patient/family
6. Providing psychological and social support
7. Anticipation and preparation for emergencies/deterioration
8. Feeling of safety

### Identifying adverse events and problems in care

We also sought to assess common adverse events and problems in care as an adjunct and expansion of patient ratings of the transition process. We derived these from an examination of relevant literature on adverse events in ambulatory care (e.g. [47]), discussion with clinicians and the PACT PPI panel. In the administration of the PACT-M we planned to ask about the presence or absence of these problems in the relevant time period and, if they had occurred, ask follow up questions to explore in more detail.

The final list of adverse events and problems in care was:

1. Having an infection (wound infection, catheter, bladder etc.)
2. Having experienced falls
3. Not being able to get a GP or other healthcare practitioner appointment
4. Problems with medication
5. Having to wait a long time for important healthcare supplies (pads, commodes, special diets etc.)
6. Having visited the emergency department or having been readmitted to hospital since their previous admission at the time of recruitment to the study

### Stage 2: Item pool generation

#### Initial item pool

Four or five questionnaire items were developed for each of the 8 components and designed to be applicable for aspects of care transition. In total, 76 items were mapped to the eight components: half (*n* = 38) related to patient’s experience of care in hospital and the remaining items related to patient’s experience of managing care at home.

### Item review and refinement

Members of the PACT team reviewed the original item pool of 76 items in roundtable discussions and ensured that the items spoke directly to the conceptual thinking that underpinned the intervention development particularly in relation to patient involvement. The team commented on the component suitability and representation of components by the measure items. The review team harmonised the item wording over time to ensure that phrasing of experience at the point of discharge at Time 1 (e.g. “Before leaving the hospital I was confident I understood how to manage my medication”) was consistent with management of care at home at Time 2 and Time 3 (e.g. “I know how to manage my medicines.”). We aimed to achieve some translation between versions of the measure in order to facilitate analysis of associations across the three time points of data collection.

### Assessing language and comprehension

To verify the level of comprehension of the questionnaire items, instructions, suitability of question format and ease of questionnaire scoring on face value, we consulted 5 members of the PPI panel in person and by telephone. For all advisory groups and consultations we did not carry out full qualitative analysis but systematically reviewed the feedback we received. Panel members reported that, on the whole, wording of questions appropriately resonated with their own experience with transitions, the rating system was clear and they would be willing to complete the PACT-M. Participants identified some potential barriers to completion of the PACT-M such as people having experienced more than one admission since their last discussion with the researcher and added minor comments on the wording of individual items. They suggested that the duration of the phone call should not exceed 30 minutes with the maximum number of questions to be 15. This feedback led to changes to the PACT –M, including adaptations to some of the wording and where necessary, revisions to items.

### Stage 3: Prioritisation of items for inclusion in the measure; a modified Delphi method

#### Delphi method and formal prioritisation of items

In each of the two successive rounds, experts reviewed the proposed items and rated them on a 4-point Likert scale ranging from ‘definitely exclude’ to ‘definitely include’. Participants could also suggest that an item be reworded, comment on the classification of items under components and recommend additional items for each component. Items that that received a “definitely Include” rating from 70% or more of the experts were accepted for inclusion in the measure and were not included in the following rounds.

Items that received a “Definitely Include” or “Probably Include’” rating from less than 50% of the experts were removed, with those higher than 50% retained for the second round. After each round, questions were modified and reworded based on the qualitative input from the experts. Recirculation ceased when the expert panel reach a general consensus.

### Final form of the PACT-M for pilot testing

The final measure administered within a week of discharge (PACT-M1) and consistsed of: (i) eight items reflecting the eight measure components around information and support patients received before and at the point of discharge, (ii) six questions around potential issues with participants’ healthcare and (iii) two open ended questions prompting participants to provide any additional piece of information they wish to disclose. The six questions about health issues and the open ended questions are repeated across the three different time points.

In later studies we plan to administer a parallel measure (PACT-M2) at Times 2 and 3 (one month and three months post-discharge respectively) that contains eight items rated on 5-point Likert scales measuring patients’ perceptions of managing their care at home. Both PACT-M1 and PACT-M2 are shown in the Additional file 1 but only the initial pilot testing of usability and acceptance of PACT-M1 is reported here. Assessment of the measure at different time points will be described in a subsequent paper.

### Stage 4: Pilot testing of the PACT-M

**Fig. 3:**
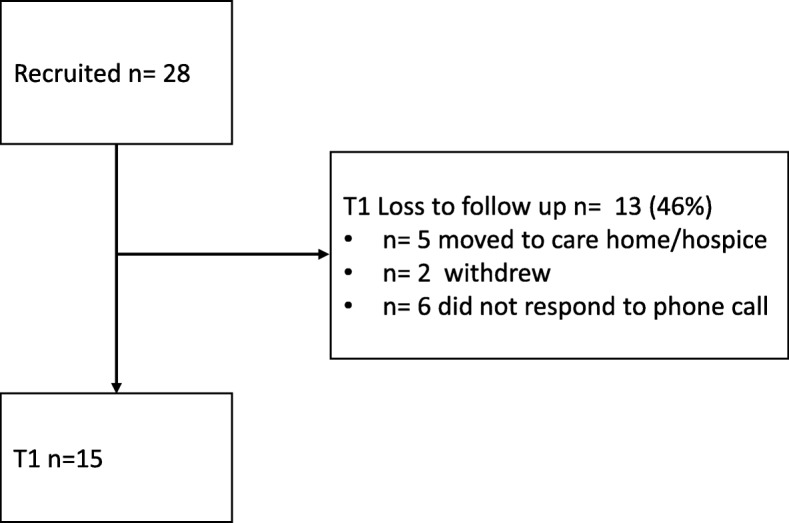
Recruitment flowchart

Of the twenty –eight recruited participants, thirteen dropped out and either became not eligible as they moved to care home/hospices, withdrew from the study or did not respond when we contacted their preferred telephone number (Fig. 3). Contact was made with 15 participants (4 female, 11 male) between three and seven days post discharge with 70% of participants having been contacted between three to five days post discharge. All participants identified themselves as of white British origin. Nine people were aged between 65 to 75, and 6 were over 75 years of age. The majority (13 people) had been discharged from the complex medical care unit and two from surgical services. Ten people lived with a spouse or partner, one with a family member and four lived alone.

### Participants’ views on the PACT-M delivery and content

#### Analysis and results

Analyses of the PACT-M quantitative responses were undertaken using IBM SPSS Statistics 25 Software Package [48]. Descriptive statistics and frequencies were calculated for each questionnaire item as well as for demographic characteristics.

Simple analysis of the content of text-based responses was used to get a sense of the prevalence of particular types of problems when completing the questionnaire and with transitions. Responses were summarized and compared across respondents for each item, noting similarities, differences, and frequencies of types of responses. Recommendations were made for item revisions and/or adjustments to the administration process.

(i) Experience of responding to the measure

Participants said they found the measure easy to respond to and “straightforward” in presentation with no difficulties reported in understanding individual items. Phone calls were shorter for people who had had an uncomplicated transition and longer for those with more complex problems or who were living alone.

(ii) Experience of transition

Participants perceived the 5-point response scale as relatively easy to use and, once understood, mostly replied with a number e.g. ‘4’, rather than agreement scale e.g. ‘I agree’. Some participants requested the response scale options to be repeated and the difference between ‘agree’ and ‘strongly agree’ was often unclear.

As seen on Table 1, the great majority of people (80%) were satisfied with the preparation for discharge and 93% agreed that they knew what to do if their health deteriorated at home. A total of 14% of participants, reported feeling they were not able to ask questions about their care while in hospital and expressed concerns about being ready to manage medication on their return home. Participants with families or partners acting as carers rated questions on further support at home as not relevant, whereas the same questions were ranked high on the agreement scale by participants who lived alone.

**Table 1:** Frequencies of the PACT-M Time 1 responses

		Strongly disagree	Disagree	Neither agree nor disagree	Agree	Strongly agree	Not applicable
1. I felt I could ask staff questions about what will happen after going home.	%	0	7	0	47	47	0
2. Before leaving the hospital I was confident I understood how to manage my medication.	%	0	7	0	47	47	0
3. While I was in hospital, staff helped me to prepare for things that I might find difficult when I go back home.	%	0	0	0	47	33	20
4. Before leaving the hospital, I understood how to get help from my community services.	%	0	0	0	33	33	33
5. Before leaving the hospital I knew what arrangements had been made to support me at home.	%	0	0	0	27	27	47
6. While I was in hospital, there was someone who I could talk to if I was worried.	%	0	0	0	40	27	33
7. Before leaving the hospital, I felt confident about what to do if my health became worse at home.	%	0	0	7	53	40	0
8. I feel that my concerns around my health had been addressed before I went home.	%	0	7	0	60	33	0

### Problems experienced by patients

Six out of fifteen participants reported having some problems managing care since their return home. The most frequent problems were: experiencing delays receiving healthcare supplies (such as catheters), requiring additional care that they had not expected or been prepared for (such as using the emergency services), or being readmitted to hospital. Although participants were instructed to comment on potential adverse events since their latest admission to hospital, (e.g. “Thinking about the last time you have been into hospital, have you had an infection?) seven people replied with a story about a health issue without clarifying whether this was from their most recent admission or a past event. Table 2 shows illustrative quotes from participants’ views on their transitional care experiences as they elaborated while responding on the Likert scale. The quotations illustrate the fact that people may give high overall ratings of their experience of transition but still report some problems in their care and some experiences of loss of independence or dignity.

**Table 2:** Verbatim quotations from participants illustrating their experience of transition

Themes	Quotes
Patient Involvement	“*I came out of the operation and didn’t have much bruising but later on I got really bruised, like 6 in. of bruising, but I didn’t mention it. The hospital didn’t tell me why so I asked my sister who is a GP. This was a normal thing, they should know that it happens. I left the day after the operation. It was busy so I didn’t have much information, I wanted to ask some questions but I couldn’t.”*
Medication management	*“I’m used to being independent but they are treating me like a kid, my condition doesn’t affect my ability to take pills. Sometimes I had to sign a form to change my medication but didn’t know why they would change the order without asking me. When I went home I didn’t really know what they changed and the medicines made me drowsy. I just think that someone like me, reasonably fit, should be allowed to dispense their own medicines.”*
Discharge arrangements	*“Some people were arrogant, me and my wife have 30 years’ experience with my condition and sometimes people are very busy and do not listen to me, I’m treated like a child.*
Providing psychological support	*”I saw a lady being informed that she had cancer and she was on her own, no family member was there. And when they asked if she had any questions she said no, but clearly she didn’t know what was happening. I personally talked to her nurse and requested her clinical staff to talk to her and her family more about her condition and the cancer.”*
Feeling of safety	*“I didn’t have the information I needed about what to do at home. I live alone and I had stomach pain, abdominal pain, I had heart problems and have been to the emergency department three times since we spoke”.*

## Discussion

This paper has described the comprehensive development process and pilot testing of the PACT-M. The PACT-M is, to our knowledge, the first instrument to incorporate a broad range of care transition components across different points of discharge and provide a comprehensive assessment for the quality and safety of moving from hospital to home, relevant to UK population.

We developed the PACT-M to evaluate the quality of transitions from hospital to home from the perspective of older patients, across 3 time points of the discharge period to address key components of discharge and post-discharge care. We were particularly concerned to look beyond communication and information transfer [49] and also to capture patient experience of being prepared for discharge and their subsequent experience of managing at home with a focus on factors underpinning patient safety. We have focussed on developing a measure that will be suitable for use with the most vulnerable patient groups such as older people with multiple comorbidities [40].

We did not find any validated measures that assess transitional quality and safety at multiple time points post discharge. A recently developed measure by Boge et al. [50] assessed 3 components of transitional care for older patients; coping after discharge, participation in discharge planning and adherence to treatment. While this survey revealed insights into patient experience of transition 30 days post discharge, it is possible that older people might not accurately recall the experience of participating in their latest discharge. Onwards, this measure does not necessarily reflect patients’ views on their latest discharge as it is possible that people could have been readmitted to the hospital before the survey administration. A recent systematic review of transitional care tools conducted by val Melle et al. [51] found 14 different instruments measuring components of patient safety of transitions and assessed them against a methodological quality tool. They found that the most important step while developing a measure is establishing content validity [51]. We have established the PACT-M’s content validity by using a modified Delphi process and by further refining the items using the PPI panel’s feedback.

Pilot testing of the PACT-M suggests that it is acceptable to older patients and that it covers issues of relevance to them. People in our patient advisory panel and those in our initial pilot testing found the measure to be straightforward and the questions relevant to their experience of being discharged home from hospital. Capturing qualitative accounts of patients’ experiences further informed the measure components and highlighted areas that other measures overlooked, such as patient safety and patient involvement in their care, post discharge. Our iterative measure development process incorporating feedback from advisory groups with our PPI panel and patient feedback, ensured that the PACT –M addresses all key aspects of transition. We believe that the long and rigorous development process is justified precisely because transitions are times of particular vulnerability for patients. We need a valid and reliable measure of transition to ensure that interventions to improve transition can reliably capture the most critical features of transition as experienced by patients and families. This instrument could also be of particular use to health care organisations seeking a practical tool to assess patient involvement and preparation for managing their own care at home.

PACT-M has been developed in the context of the UK healthcare system and is designed to work effectively in that setting. The precise arrangements for transitions from hospital to home and the nature and extent of community care and support vary widely from country to country. However, we believe that the areas addressed in the PACT-M are generic and potentially relevant to any healthcare system. PACT-M would of course have to be piloted and tested if used in another country and might require adjustments to wording and language. However, in principle it could be used in any healthcare system that manages transitions between hospital and home.

This research has some limitations. Although we performed an exhaustive process of measure development, the sample we tested the PACT-M primarily consisted of patients of white British ethnicity, mostly living with their partners, and from surgical and complex medical care units. This implied that the pilot findings may not be applicable to a more diverse sample or to patients discharged from other units such as cardiothoracic, geriatric, vascular, etc. It is also important to note that the PACT-M is only appropriate for use by patients with cognitive capacity to consent. At the present time, we consider that the PACT-M shows promise but it cannot yet be recommended for routine use. However, sufficient information is provided in this paper so that other researchers can consider testing the PACT-M with a wider population.

Much remains to be done to assess the use of PACT-M in practice and to determine its reliability and validity as a measure of patient experience of care transitions. The next phase of this measure development will involve the recruitment of 150 patients over 65 years of age being discharged from various wards. The PACT-M will be administered over the phone or by post at three time points post discharge. This will provide additional information on the structure of the measure, the relationship between items and on the relationship between the scores at different time points. We will also gain a fuller understanding of the experience of transition for older patients discharged from English hospitals.

Developing an acceptable, valid and reliable measure is essential to the evaluation of interventions to improve transitions in care and this is more likely to be achieved if future studies consider testing the measure widely, involving different groups of clinicians and researchers.

## Conclusion

We used an established four-stage procedure to develop a measure to assess the quality of care transitions from the perspective of older people. This is the first transitions measure developed specifically for UK population. The pilot testing of the PACT-M supports the usability and face validity of the PACT-M for measuring patient perceptions of factors central to safety of transitional care namely: patient involvement, information sharing and medication management. The measure components could be of value for identifying problems in the immediate post-discharge period as well as in the longer term. Such information could be useful to those involved in planning discharge care and for hospitals who want to improve the safety and continuity of care of their services for older patients. Further work is needed to explore the psychometric characteristics of the tool. We are currently testing the measure in large measure validation study which we will discuss in a future report.

## Additional file

Additional file 1: PACT-M 1 Pilot Version. (pdf 166 kb)

## Data Availability

The datasets used and analysed during the current study are available from the corresponding author on reasonable request.

## Abbreviations

CTM: Care Transitions Measure
GP: General Practitioner
NHS: National Health Service
PACT: Partners at Care Transitions
PACT-M: Partners at Care Transitions Measure
PPI: Patient and Public Involvement
