# Supplementary material for: Developing a measure to assess the quality of care transitions for older people
Source: BMC Health Serv Res. 2019 Jul 19;19:505. doi: 10.1186/s12913-019-4306-8 (PMC6642522; doi:10.1186/s12913-019-4306-8)
Supplement: Supplementary file 1 — PACT-M 1 Pilot Version. (pdf 166 kb) [file 12913_2019_4306_MOESM1_ESM.pdf]

Additional file 1

PACT-M 1 Pilot Version

	Strongly Disagree	Disagree	Neither Agree nor Disagree	Agree	Agree Strongly	Don't Know/Don't Remember/ Not Applicable
1. I felt I could ask staff questions about what will happen after going home.						
2. Before leaving the hospital I was confident I understood how to manage my medication.						
3. While I was in hospital, staff helped me to prepare for things that I might find difficult when I go back home.						
4. Before leaving the hospital, I understood how to get help or support from my community services.						
5. Before leaving the hospital I knew what arrangements had been made to support me at home.						
6. While II was in hospital, there was someone who I could talk to if I was worried about going home.						
7. Before leaving the hospital, I felt confident about what to do if my health became worse at home.						
8. I feel that my concerns around my health had been addressed before I went home.						

Problems in your care	Yes	No
1. Have you had any infections?		
2. Have you had a fall?		
3. Have you had any difficulty getting an appointment with a doctor or other healthcare person?		
4. Have you had any problems with your medication?		
5. Have you had any problems getting essential healthcare supplies (like pads or prescribed feed)?		
6. Have you had any additional problems that led to contacting the GP or anyone else, or go to the hospital or emergency services?		

If you have answered yes to any of the above questions, could you please tell us more?

Open text question	Free text
What else would you like to add?	

PACT-M 2 Pilot Version

	Strongly Disagree	Disagree	Neither Agree nor Disagree	Agree	Agree Strongly	Don't Know/Don't Remember/Not Applicable
1. I know who to contact if I have any questions around my health and healthcare.						
2. I know how to manage my medicines.						
3. I have the necessary support to manage everyday activities (e.g. cooking, cleaning, buying food, showering, walking, dressing).						
4. I feel I have the support I need from community health services (e.g. doctors, nurses, home care staff).						
5. I feel confident about managing my health at home.						
6. I feel that there is someone I can talk to about my worries (for example, health care staff or my family).						
7. I know what to do and who to contact if my health gets worse.						
8. I feel I can now manage my care safely at home						

**Problems in your care**	**Yes**	**No**
1. Have you had any infections?		
2. Have you had a fall?		
3. Have you had any difficulty getting an appointment with a doctor or other healthcare person?		
4. Have you had any problems with your medication?		
5. Have you had any problems getting essential healthcare supplies (like pads or prescribed feed)?		
6. Have you had any additional problems that led to contacting the GP or anyone else, or go to the hospital or emergency services?		

If you have answered yes to any of the above questions, could you please tell us more?

Open text question	Free text
What else would you like to add?	
